# The genome sequence of the Chevron, *Eulithis testata* (Linnaeus, 1761)

**DOI:** 10.12688/wellcomeopenres.19433.1

**Published:** 2023-05-18

**Authors:** David C. Lees, Natural History Museum Genome Acquisition Lab, Darwin Tree of Life Barcoding collective, Wellcome Sanger Institute Tree of Life programme, Wellcome Sanger Institute Scientific Operations: DNA Pipelines collective, Tree of Life Core Informatics collective, Darwin Tree of Life Consortium

**Affiliations:** 1 Natural History Museum, London, England, UK

**Keywords:** Eulithis testata, Chevron, genome sequence, chromosomal, Lepidoptera

## Abstract

We present a genome assembly from an individual male *Eulithis testata* (the Chevron; Arthropoda; Insecta; Lepidoptera; Geometridae). The genome sequence is 308.1 megabases in span. Most of the assembly is scaffolded into 30 chromosomal pseudomolecules, including the Z sex chromosome. The mitochondrial genome has also been assembled and is 15.9 kilobases in length. Gene annotation of this assembly on Ensembl identified 16,167 protein coding genes.

## Species taxonomy

Eukaryota; Metazoa; Ecdysozoa; Arthropoda; Hexapoda; Insecta; Pterygota; Neoptera; Endopterygota; Lepidoptera; Glossata; Ditrysia; Geometroidea; Geometridae; Larentiinae; *Eulithis*; *Eulithis testata* (Linnaeus, 1761) (NCBI:txid326959).

## Background

**Figure 1.**
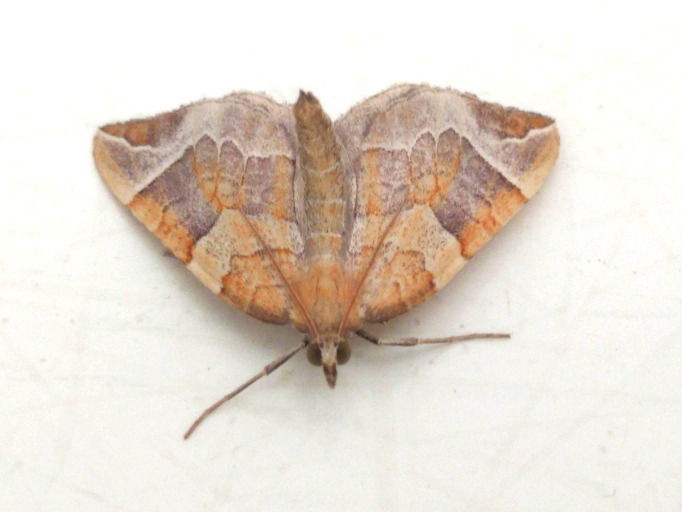
Photograph of *Eulithis testata*. https://www.inaturalist.org/photos/97801019 (c) Michel Langeveld, some rights reserved (CC BY-SA).

The Chevron, *Eulithis testata*, is a medium sized geometrid moth, quite variable in ground colour, often with a beautiful mixture of yellowish scales frosted with grey on the forewing, distinguished by a series of six strongly angled chevron markings following one another, and a somewhat semicircular brown blotch at the termen ( Figure 1). The Chevron emerges in late June or July and flies (at dusk) until mid-September in the UK ( Randle *et al.*, 2019), overwintering as an egg.

The Chevron is found in a range of habitats especially moorland and heathland, also marshes and sand dunes ( Randle *et al.*, 2019). Its larval foodplants comprise mainly different shrubs particularly *Salix* species, as well as birch and heather further north ( Waring *et al.*, 2017).

*E. testata* is one of a few geometrid moths that span the Holarctic ( GBIF Secretariat, 2022), and it is widespread throughout the United Kingdom ( NBN Atlas Partnership, 2021). Populations in the UK show a significant decrease since 1970 in both abundance and distribution ( Conrad *et al.*, 2006; Randle *et al.*, 2019).

**Figure 2.**
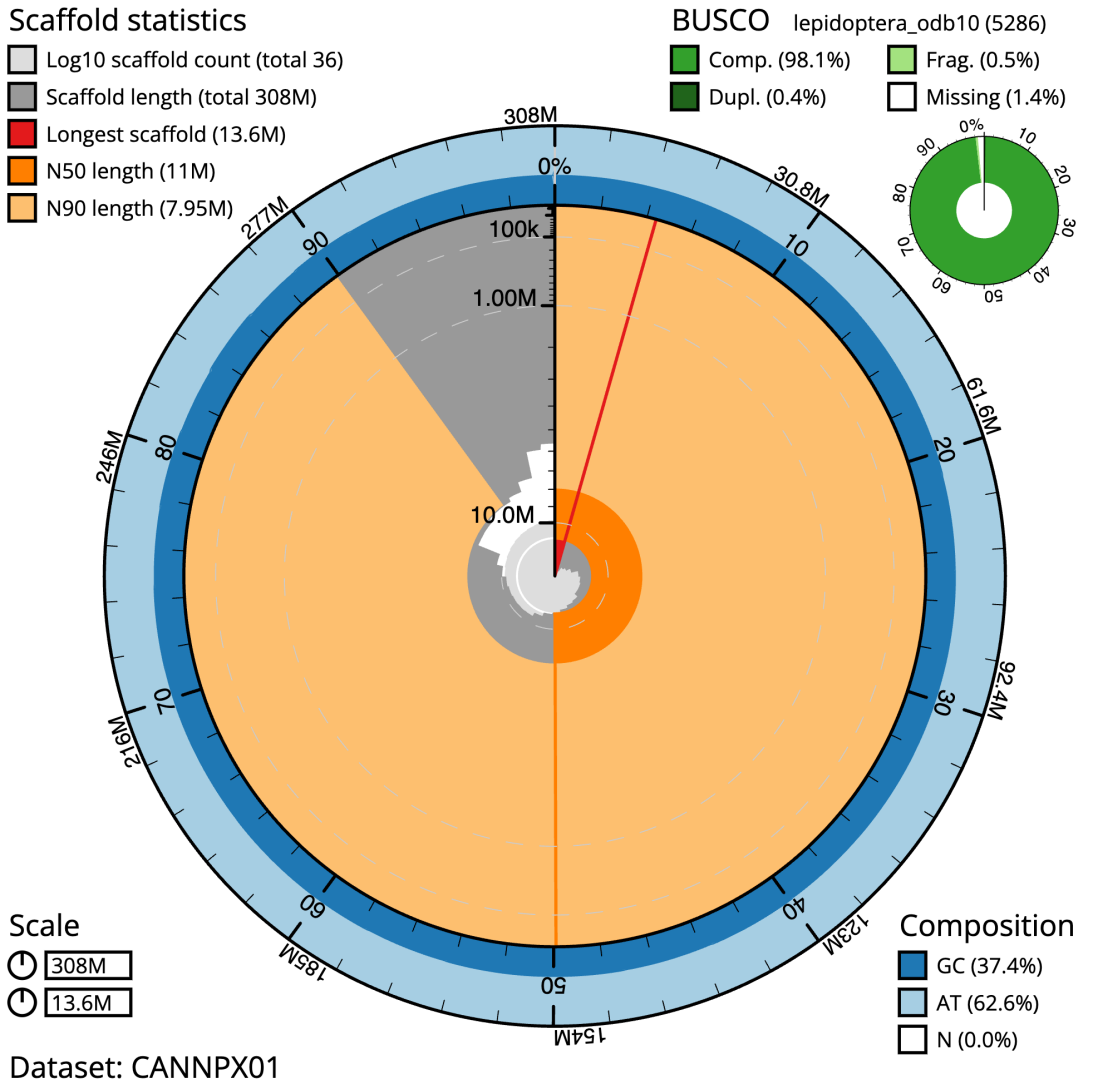
Genome assembly of *Eulithis testata*, ilEulTest2.1: metrics. The BlobToolKit Snailplot shows N50 metrics and BUSCO gene completeness. The main plot is divided into 1,000 size-ordered bins around the circumference with each bin representing 0.1% of the 308,098,708 bp assembly. The distribution of scaffold lengths is shown in dark grey with the plot radius scaled to the longest scaffold present in the assembly (13,628,168 bp, shown in red). Orange and pale-orange arcs show the N50 and N90 scaffold lengths (10,981,015 and 7,954,720 bp), respectively. The pale grey spiral shows the cumulative scaffold count on a log scale with white scale lines showing successive orders of magnitude. The blue and pale-blue area around the outside of the plot shows the distribution of GC, AT and N percentages in the same bins as the inner plot. A summary of complete, fragmented, duplicated and missing BUSCO genes in the lepidoptera_odb10 set is shown in the top right. An interactive version of this figure is available at https://blobtoolkit.genomehubs.org/view/Eulithis%20testata/dataset/CANNPX01/snail.

The genus *Eulithis* is currently placed in the larentiine tribe Cidariini ( Choi, 1997). It is sister to the genus *Ecliptopera* in the study of Õunap, *et al*. ( 2016; Figure 2). Evolution of its wing patterns, including those of its many congeners have been studied, and one species *Eulithis convergenata* (Bremer, 1864) appears to be mimetic of the aposematic geometrid genus *Abraxas* ( Choi, 2001).

The genome sequence should not only be useful in phylogeny, but also in studies of potentially cryptic species. There are two DNA barcode clusters on BOLD (17 April 2023), the BINs BOLD:AAB0154 and BOLD:ABZ0682 (the former in the USA and Canada, the latter in the Palaearctic including the UK and Europe), and these two discrete clusters are only about 1.47% pairwise divergent from each other.

The genome of *Eulithis testata* was sequenced as part of the Darwin Tree of Life Project, a collaborative effort to sequence all named eukaryotic species in the Atlantic Archipelago of Britain and Ireland. Here we present a chromosomally complete genome sequence for *Eulithis testata*, based on one male specimen from Beinn Eighe National Nature Reserve, Scotland, UK.

## Genome sequence report

The genome was sequenced from one male *Eulithis testata*. A total of 60-fold coverage in Pacific Biosciences single-molecule HiFi long reads was generated. Primary assembly contigs were scaffolded with chromosome conformation Hi-C data. Manual assembly curation corrected 12 missing joins or mis-joins, reducing the scaffold number by 12.2%.

The final assembly has a total length of 308.1 Mb in 36 sequence scaffolds with a scaffold N50 of 11.0 Mb ( Table 1). Most (99.92%) of the assembly sequence was assigned to 28 chromosomal-level scaffolds, representing 30 autosomes and the Z sex chromosome. Chromosome-scale scaffolds confirmed by the Hi-C data are named in order of size ( Figure 2– Figure 5; Table 2). While not fully phased, the assembly deposited is of one haplotype. Contigs corresponding to the second haplotype have also been deposited. The mitochondrial genome was also assembled and can be found as a contig within the multifasta file of the genome submission.

**Table 1.** Genome data for *Eulithis testata*, ilEulTest2.1.

Project accession data		
Assembly identifier	ilEulTest2.1	
Species	*Eulithis testata*	
Specimen	ilEulTest2	
NCBI taxonomy ID	326959	
BioProject	PRJEB55881	
BioSample ID	SAMEA14448148	
Isolate information	ilEulTest2, male; head and thorax (genome sequencing) ilEulTest3, head and thorax (Hi-C scaffolding)	
Assembly metrics *		*Benchmark*
Consensus quality (QV)	67.7	*≥ 50*
*k*-mer completeness	100%	*≥ 95%*
BUSCO **	C:98.1%[S:97.8%,D:0.4%], F:0.5%,M:1.4%,n:5,286	*C ≥ 95%*
Percentage of assembly mapped to chromosomes	99.92%	*≥ 95%*
Sex chromosomes	Z chromosome	*localised homologous pairs*
Organelles	Mitochondrial genome assembled	*complete single alleles*
Raw data accessions		
PacificBiosciences SEQUEL II	ERR10224850	
Hi-C Illumina	ERR10177756	
Genome assembly		
Assembly accession	GCA_947507515.1	
*Accession of alternate haplotype*	GCA_947507505.1	
Span (Mb)	308.1	
Number of contigs	69	
Contig N50 length (Mb)	9.3	
Number of scaffolds	36	
Scaffold N50 length (Mb)	11.0	
Longest scaffold (Mb)	13.6	
Genome annotation		
Number of protein-coding genes	16,167	
Number of gene transcripts	16,359	

* Assembly metric benchmarks are adapted from column VGP-2020 of “Table 1: Proposed standards and metrics for defining genome assembly quality” from ( Rhie *et al.*, 2021). ** BUSCO scores based on the lepidoptera_odb10 BUSCO set using v5.3.2. C = complete [S = single copy, D = duplicated], F = fragmented, M = missing, n = number of orthologues in comparison. A full set of BUSCO scores is available at https://blobtoolkit.genomehubs.org/view/Eulithis%20testata/dataset/CANNPX01/busco.

**Figure 3.**
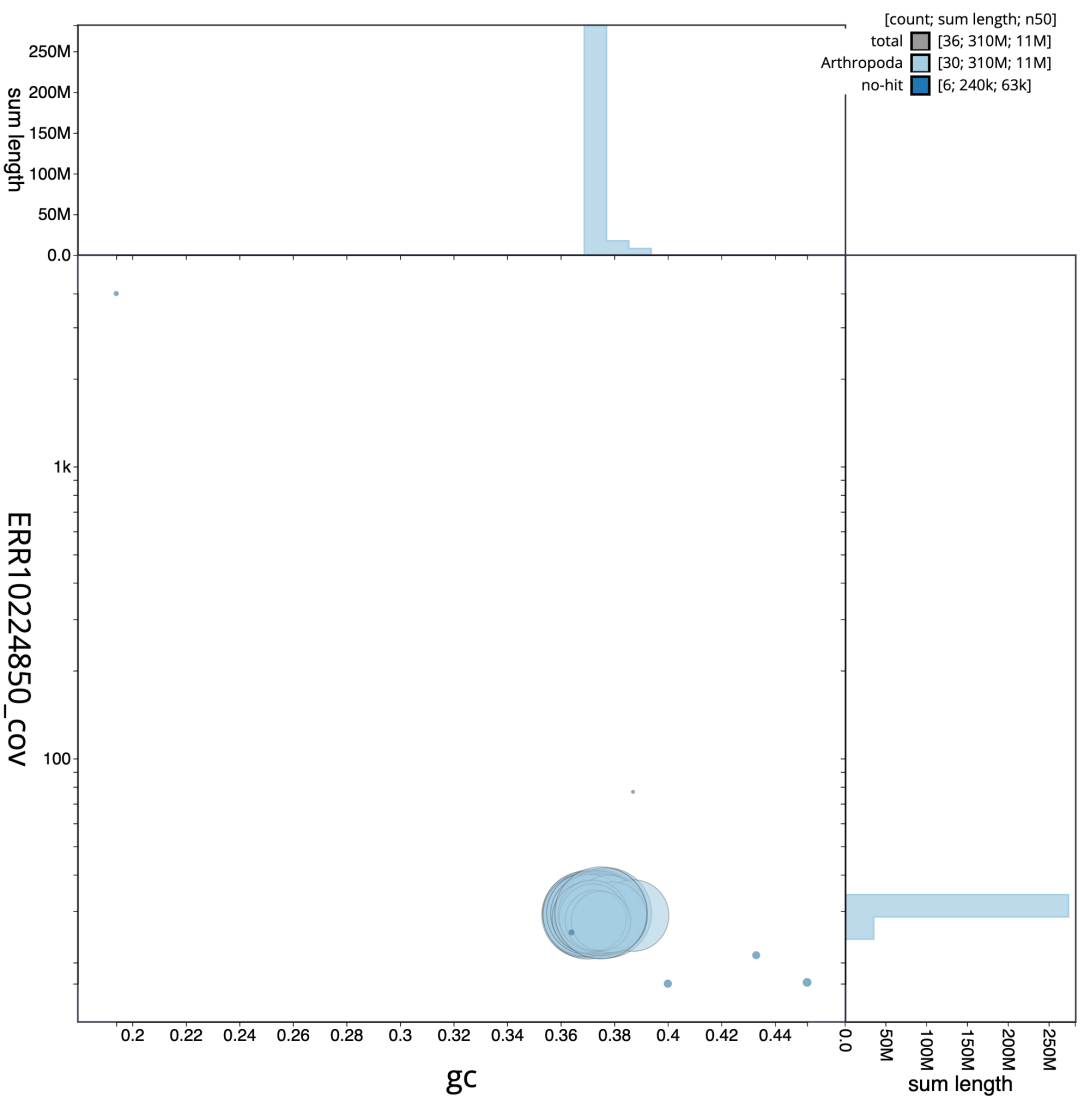
Genome assembly of *Eulithis testata*, ilEulTest2.1: BlobToolKit GC-coverage plot. Scaffolds are coloured by phylum. Circles are sized in proportion to scaffold length. Histograms show the distribution of scaffold length sum along each axis. An interactive version of this figure is available at https://blobtoolkit.genomehubs.org/view/Eulithis%20testata/dataset/CANNPX01/blob.

**Figure 4.**
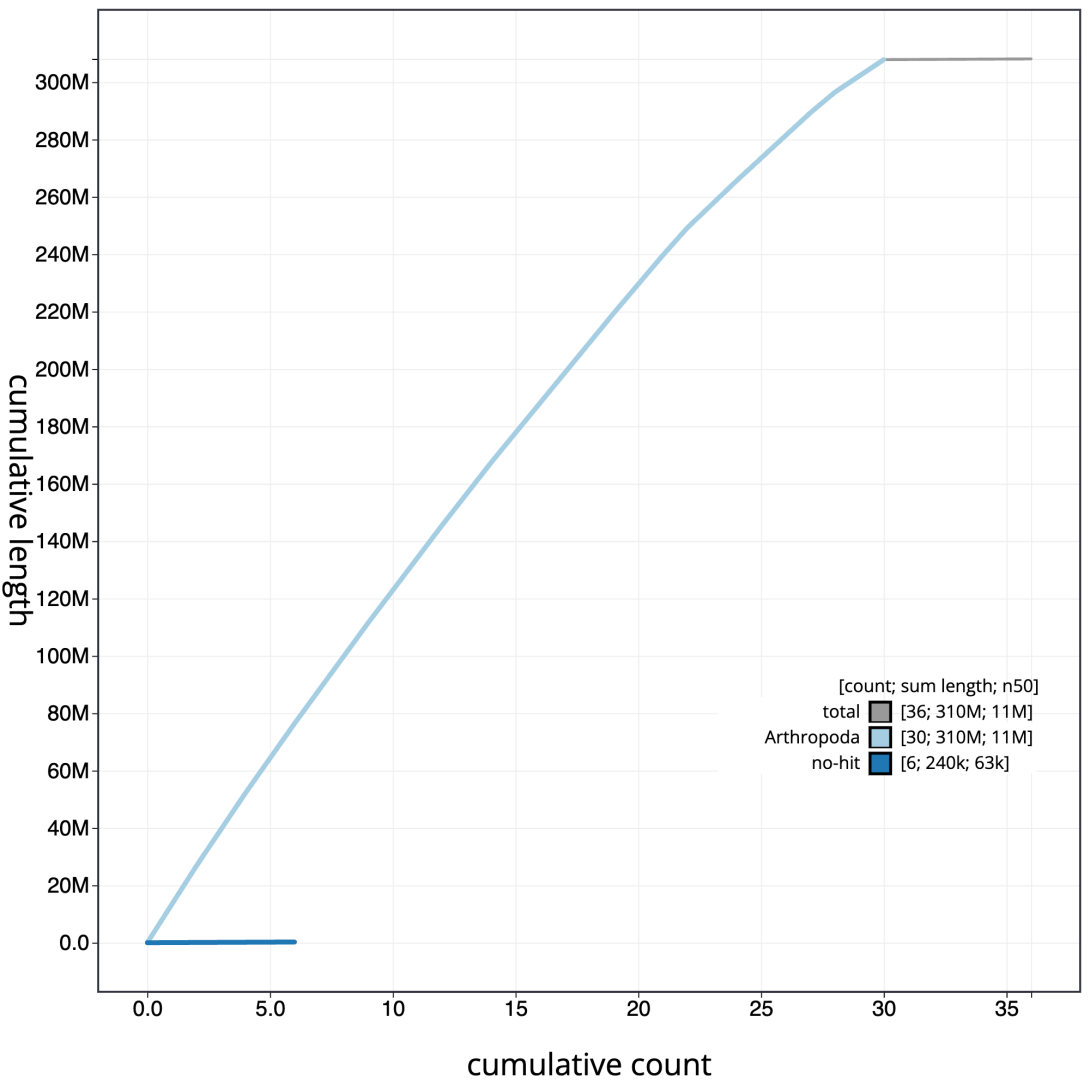
Genome assembly of *Eulithis testata*, ilEulTest2.1: BlobToolKit cumulative sequence plot. The grey line shows cumulative length for all scaffolds. Coloured lines show cumulative lengths of scaffolds assigned to each phylum using the buscogenes taxrule. An interactive version of this figure is available at https://blobtoolkit.genomehubs.org/view/Eulithis%20testata/dataset/CANNPX01/cumulative.

**Figure 5.**
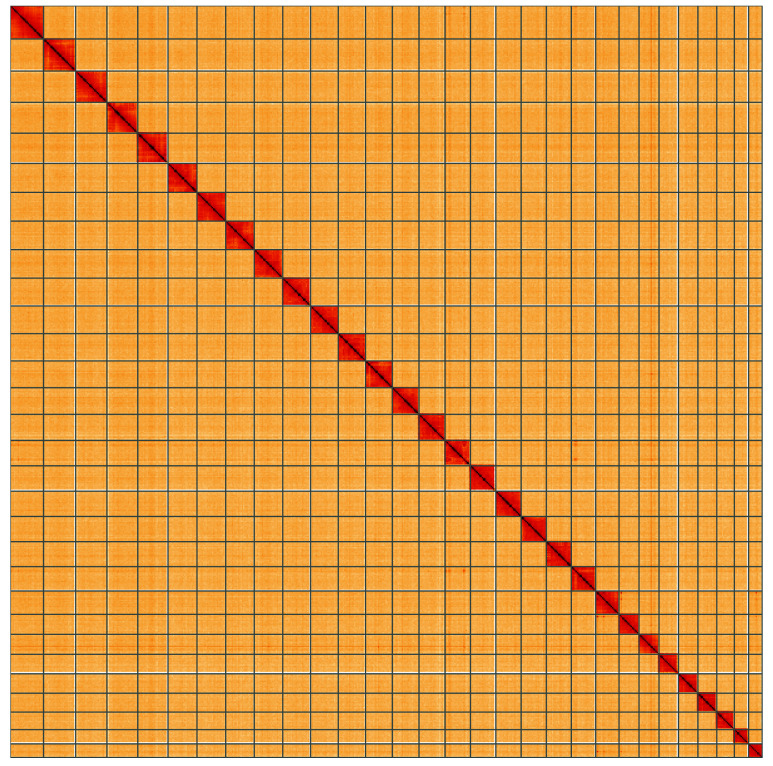
Genome assembly of *Eulithis testata*, ilEulTest2.1: Hi-C contact map of the ilEulTest2.1 assembly, visualised using HiGlass. Chromosomes are shown in order of size from left to right and top to bottom. An interactive version of this figure may be viewed at https://genome-note-higlass.tol.sanger.ac.uk/l/?d=dYxE9IdaTNu__uKX_RLc4g.

**Table 2.** Chromosomal pseudomolecules in the genome assembly of *Eulithis testata*, ilEulTest2.

INSDC accession	Chromosome	Size (Mb)	GC%
OX382194.1	1	13.13	37.7
OX382195.1	2	12.79	37.4
OX382196.1	3	12.65	37.5
OX382197.1	4	12.36	37.6
OX382198.1	5	11.88	36.9
OX382199.1	6	11.76	37.1
OX382200.1	7	11.68	37
OX382201.1	8	11.67	37.1
OX382202.1	9	11.41	36.9
OX382203.1	10	11.33	37.5
OX382204.1	11	11.16	36.9
OX382205.1	12	10.98	37.3
OX382206.1	13	10.9	37.4
OX382207.1	14	10.66	37
OX382208.1	15	10.41	37.5
OX382209.1	16	10.39	37.5
OX382210.1	17	10.37	37.3
OX382211.1	18	10.31	37.4
OX382212.1	19	10.23	37.6
OX382213.1	20	9.97	37.1
OX382214.1	21	9.57	37.8
OX382215.1	22	8.19	37.1
OX382216.1	23	8.17	38.7
OX382217.1	24	7.96	37
OX382218.1	25	7.95	37.9
OX382219.1	26	7.73	37.2
OX382220.1	27	7.18	37.2
OX382221.1	28	5.84	37.3
OX382222.1	29	5.58	37.5
OX382193.1	Z	13.63	37.5
OX382223.1	MT	0.02	19.4
-	unplaced	0.23	42.2

The estimated Quality Value (QV) of the final assembly is 67.7 with *k*-mer completeness of 100%, and the assembly has a BUSCO v5.3.2 completeness of 98.1% (single = 97.8%, duplicated = 0.4%), using the lepidoptera_odb10 reference set ( *n* = 5,286).

Metadata for specimens, spectral estimates, sequencing runs, contaminants and pre-curation assembly statistics can be found at https://links.tol.sanger.ac.uk/species/326959.

## Genome annotation report

The *E. testata* genome assembly (GCA_947507515.1) was annotated using the Ensembl rapid annotation pipeline ( Table 1; https://rapid.ensembl.org/Eulithis_testata_GCA_947507515.1/Info/Index). The resulting annotation includes 16,359 transcribed mRNAs from 16,167 protein-coding genes.

## Methods

### Sample acquisition and nucleic acid extraction

Two *Eulithis testata* specimens (ilEulTest2 and ilEulTest3) were collected from Beinn Eighe National Nature Reserve, Scotland, UK (latitude 57.63, longitude –5.35) on 10 September 2021 using a light trap. The specimens were collected and identified by David Lees (Natural History Museum) and dry frozen at –80°C. Individual ilEulTest2 (specimen no. NHMUK014543798) was used for genome sequencing, while individual ilEulTest3 (specimen no. NHMUK014543792) was used for Hi-C scaffolding.

DNA was extracted at the Tree of Life laboratory, Wellcome Sanger Institute (WSI). The ilEulTest2 sample was weighed and dissected on dry. Head and thorax tissue was disrupted using a Nippi Powermasher fitted with a BioMasher pestle. High molecular weight (HMW) DNA was extracted using the Qiagen MagAttract HMW DNA extraction kit. HMW DNA was sheared into an average fragment size of 12–20 kb in a Megaruptor 3 system with speed setting 30. Sheared DNA was purified by solid-phase reversible immobilisation using AMPure PB beads with a 1.8X ratio of beads to sample to remove the shorter fragments and concentrate the DNA sample. The concentration of the sheared and purified DNA was assessed using a Nanodrop spectrophotometer and Qubit Fluorometer and Qubit dsDNA High Sensitivity Assay kit. Fragment size distribution was evaluated by running the sample on the FemtoPulse system.

### Sequencing

Pacific Biosciences HiFi circular consensus DNA sequencing libraries were constructed according to the manufacturers’ instructions. DNA sequencing was performed by the Scientific Operations core at the WSI on the Pacific Biosciences SEQUEL II instrument. Hi-C data were also generated from head and thorax tissue of ilEulTest3 using the Arima2 kit and sequenced on the Illumina NovaSeq 6000 instrument.

### Genome assembly, curation and evaluation

Assembly was carried out with Hifiasm ( Cheng *et al.*, 2021) and haplotypic duplication was identified and removed with purge_dups ( Guan *et al.*, 2020). The assembly was then scaffolded with Hi-C data ( Rao *et al.*, 2014) using YaHS ( Zhou *et al.*, 2023). The assembly was checked for contamination as described previously ( Howe *et al.*, 2021). Manual curation was performed using HiGlass ( Kerpedjiev *et al.*, 2018) and Pretext ( Harry, 2022). The mitochondrial genome was assembled using MitoHiFi ( Uliano-Silva *et al.*, 2022), which runs MitoFinder ( Allio *et al.*, 2020) or MITOS ( Bernt *et al.*, 2013) and uses these annotations to select the final mitochondrial contig and to ensure the general quality of the sequence.

A Hi-C map for the final assembly was produced using bwa-mem2 ( Vasimuddin *et al.*, 2019) in the Cooler file format ( Abdennur & Mirny, 2020). To assess the assembly metrics, the *k*-mer completeness and QV consensus quality values were calculated in Merqury ( Rhie *et al.*, 2020). This work was done using Nextflow ( Di Tommaso *et al.*, 2017) DSL2 pipelines “sanger-tol/readmapping” ( Surana *et al.*, 2023a) and “sanger-tol/genomenote” ( Surana *et al.*, 2023b). The genome was analysed within the BlobToolKit environment ( Challis *et al.*, 2020) and BUSCO scores ( Manni *et al.*, 2021; Simão *et al.*, 2015) were calculated.

Table 3 contains a list of relevant software tool versions and sources.

**Table 3.** Software tools: versions and sources.

Software tool	Version	Source
BlobToolKit	4.0.7	https://github.com/blobtoolkit/blobtoolkit
BUSCO	5.3.2	https://gitlab.com/ezlab/busco
Hifiasm	0.16.1-r375	https://github.com/chhylp123/hifiasm
HiGlass	1.11.6	https://github.com/higlass/higlass
Merqury	MerquryFK	https://github.com/thegenemyers/MERQURY.FK
MitoHiFi	2	https://github.com/marcelauliano/MitoHiFi
PretextView	0.2	https://github.com/wtsi-hpag/PretextView
purge_dups	1.2.3	https://github.com/dfguan/purge_dups
sanger-tol/genomenote	v1.0	https://github.com/sanger-tol/genomenote
sanger-tol/readmapping	1.1.0	https://github.com/sanger-tol/readmapping/tree/1.1.0
YaHS	yahs-1.1.91eebc2	https://github.com/c-zhou/yahs

### Genome annotation

The BRAKER2 pipeline ( Brůna *et al.*, 2021) was used in the default protein mode to generate annotation for the *Eulithis testata* assembly (GCA_947507515.1) in Ensembl Rapid Release.

### Ethics and compliance issues

The materials that have contributed to this genome note have been supplied by a Darwin Tree of Life Partner. The submission of materials by a Darwin Tree of Life Partner is subject to the Darwin Tree of Life Project Sampling Code of Practice. By agreeing with and signing up to the Sampling Code of Practice, the Darwin Tree of Life Partner agrees they will meet the legal and ethical requirements and standards set out within this document in respect of all samples acquired for, and supplied to, the Darwin Tree of Life Project. Each transfer of samples is further undertaken according to a Research Collaboration Agreement or Material Transfer Agreement entered into by the Darwin Tree of Life Partner, Genome Research Limited (operating as the Wellcome Sanger Institute), and in some circumstances other Darwin Tree of Life collaborators.

## Data Availability

European Nucleotide Archive: *Eulithis testata*. Accession number PRJEB55881; https://identifiers.org/ena.embl/PRJEB55881 ( Wellcome Sanger Institute, 2022). The genome sequence is released openly for reuse. The *Eulithis testata* genome sequencing initiative is part of the Darwin Tree of Life (DToL) project. All raw sequence data and the assembly have been deposited in INSDC databases. Raw data and assembly accession identifiers are reported in Table 1.

## Author information

Members of the Natural History Museum Genome Acquisition Lab are listed here:

https://doi.org/10.5281/zenodo.4790042.

Members of the Darwin Tree of Life Barcoding collective are listed here: https://doi.org/10.5281/zenodo.4893703.

Members of the Wellcome Sanger Institute Tree of Life programme are listed here: https://doi.org/10.5281/zenodo.4783585.

Members of Wellcome Sanger Institute Scientific Operations: DNA Pipelines collective are listed here: https://doi.org/10.5281/zenodo.4790455.

Members of the Tree of Life Core Informatics collective are listed here: https://doi.org/10.5281/zenodo.5013541.

Members of the Darwin Tree of Life Consortium are listed here: https://doi.org/10.5281/zenodo.4783558.
