# Supplementary material for: The genome sequence of the Chevron, Eulithis testata (Linnaeus, 1761)
Source: Wellcome Open Res. 2023 May 18;8:223. [Version 1] doi: 10.12688/wellcomeopenres.19433.1 (PMC11128040; doi:10.12688/wellcomeopenres.19433.1)
Supplement: Peer review history

# Reviewer report: Reviewer response for version 1

**DOI:** 10.21956/wellcomeopenres.21529.r70726

**Published:** 2024-05-25

**Authors:** Maurijn van der Zee

**Affiliations:** 1 Leiden University, Sylviusweg, Leiden, The Netherlands

David Lees provides the genome of the the butterfly *Eulithis testat*a. It is a high quality genome, based on PacBio long reads with a 60-fold coverage. This coverage is likely to eliminate sequence reading errors. Particularly powerful is that Hi-C data and manual curation reduced the scaffold number to 36, which practically equals the number of chromosomes (30 autosomes). The Busco score of 98.1% underlines the completeness of the genome. The only weakness I can think of is that annotation is only based on the ENSEMBL rapid annotation pipeline, and that no transcriptomic data have been generated and used. The genome will be useful in many research fields, including phylogeny and taxonomy.

Are sufficient details of methods and materials provided to allow replication by others?

Yes

Is the rationale for creating the dataset(s) clearly described?

Yes

Are the datasets clearly presented in a useable and accessible format?

Yes

Are the protocols appropriate and is the work technically sound?

Yes

Reviewer Expertise:

comparative genomics, evo-devo

I confirm that I have read this submission and believe that I have an appropriate level of expertise to confirm that it is of an acceptable scientific standard.

# Reviewer report: Reviewer response for version 1

**DOI:** 10.21956/wellcomeopenres.21529.r70725

**Published:** 2023-12-22

**Authors:** José María Martín-Durán

**Affiliations:** 1 Queen Mary University of London, London, England, UK

This is the genome report of the moth Eulithis testata, a common lepidopteran in the UK, now in decline. The genome assembly meets the gold-standard criteria of the Darwin Tree of Life initiative, with virtually all the genome contained in 28 chromosomal-level scaffolds. This will be a valuable resource for the community and broadly benefit the research communities working on insect conservation, ecology, and evolutionary genomics.

Are sufficient details of methods and materials provided to allow replication by others?

Yes

Is the rationale for creating the dataset(s) clearly described?

Yes

Are the datasets clearly presented in a useable and accessible format?

Yes

Are the protocols appropriate and is the work technically sound?

Yes

Reviewer Expertise:

Evolutionary developmental biology and comparative genomics

I confirm that I have read this submission and believe that I have an appropriate level of expertise to confirm that it is of an acceptable scientific standard.
